# A survey on management practices of hypotension in preterm neonates: an Indian perspective

**DOI:** 10.3389/fped.2024.1411719

**Published:** 2024-10-21

**Authors:** Rupam Das, Rema Nagpal, Sujata Deshpande, Gunjana Kumar, Anita Singh, Aditya Kallimath, Pradeep Suryawanshi

**Affiliations:** ^1^ Department of Neonatology, Bharati Vidyapeeth University Medical College, Hospital, and Research Centre, Pune, India; ^2^ Department of Neonatology, National Institute of Medical Sciences and Research, Jaipur, India; ^3^ Sanjay Gandhi Postgraduate Institute of Medical Sciences, Lucknow, India

**Keywords:** hypotension, survey, practice variations, Indian NICUs, preterm

## Abstract

**Background:**

Hypotension is a common entity in the neonatal intensive care unit (NICU) and is reported in 24%–50% of preterm infants with birth weight less than 1,500 g. Rapid diagnosis and aggressive management is crucial to reduce its detrimental effects on end-organs especially the brain. Physicians often rely on blood pressure alone as a reliable indicator of tissue perfusion, but variations exist in the definition of this crucial parameter. There are also practice variations in the use of diagnostic tools and management modalities among physicians.

**Methodology:**

A physician-based cross-sectional survey of management practices of hypotension in preterm neonates in Indian NICUs was conducted using an online survey tool. The questionnaire addressed diagnostic criteria used, utility of echocardiography for the assessment of hypotension, and management strategies used, such as volume expansion, inotropes and steroids.

**Results:**

Three hundred and twenty physicians, working predominantly in Level III NICUs, responded to the survey. The practice of delayed cord clamping was followed in the units of 78% respondents. Only 44% respondents had an institutional written protocol for the management of hypotension. The criteria for the diagnosis of hypotension varied, with 52% using mean blood pressure (BP) less than gestational age as the criteria. Capillary refill time, blood pressure and heart rate were the most common clinical criteria used. 85% respondents used echocardiography in the NICU, but only 73% utilised it for assessment of a hypotensive neonate. Physicians preferred a ‘volume-inotrope-echo-steroid’ strategy, with 85% respondents using volume expansion. Dopamine was the preferred first line inotrope, followed by norepinephrine and low-dose epinephrine.

**Conclusion:**

This survey reflects significant variations in practice amongst neonatal physicians in India. Bedside targeted echocardiography needs to be better utilised as a vital tool to determine the pathophysiology of disease and hemodynamic monitoring in the management of hypotension in neonates. While further research is needed on outcome-oriented objectives, awareness and dissemination of already existing guidelines would be useful to standardize clinical practice.

## Introduction

Hypotension is a frequently encountered entity in vulnerable preterm neonates in the neonatal intensive care unit (NICU), particularly the extremely low birth weight (ELBW), and neonates ≤28 weeks. The incidence of hypotension has been variedly reported as approximately 24%–50% in very low birth weight (VLBW) infants (1–4) and higher in ELBW neonates. This is a complex entity, with differing etiology, varying associated cardiovascular physiologies, and unpredictable outcomes. Rapid diagnosis and aggressive management of hypotension is often imperative for mitigating its detrimental effects on end-organs, particularly the brain, in the developing neonate. Blood pressure (BP) variations correlate with both the gestational and postnatal age in neonates. Postnatally, physicians often rely on blood pressure as an indicator of tissue perfusion, supplemented with clinical criteria such as capillary refill time, echocardiography and laboratory parameters.

There are tremendous controversies, with no uniform criteria for the diagnosis of hypotension, leading to significant physician and institutional variations in the management of this condition in neonates (5, 6). While many physicians use the recommendation of the Joint Working Group of the British Association of Perinatal Medicine (7) [mean arterial BP should be maintained above the gestational age (GA) in weeks] to diagnose hypotension; many others use predefined BP thresholds on centile curves for defining hypotension, varying from 3rd to 10th centiles (2, 8). However, it is crucial to acknowledge that BP is only one of the variables determining tissue perfusion.

The mainstay of treatment of hypotension in preterm neonates has, historically, included volume expansion, inotropes and vasopressors. Approximately 50% ELBW neonates receive anti-hypotensive medication (9). However, the complex hemodynamics of hypotension in preterm neonates, along with the availability of multiple inotropic agents, and absence of outcome-based evidence, leads to more questions than answers for the treating physician in choosing the right inotrope (10). Furthermore, the potential harm associated with aggressive interventions to manage hypotension, such as an elevated risk of intraventricular hemorrhage, can compound the complexity of treatment decisions (11).

In this cross-sectional study, we aimed to determine the variations of practices amongst neonatal physicians in India, in the management of hypotension in preterm neonates, by using a questionnaire-based survey. We evaluated the diagnostic criteria, determined common etiologies for hypotension, and ascertained the management modalities adopted for circulatory support in preterm neonates.

## Methodology

The survey was conducted following institutional ethics approval. A survey questionnaire, sent in early March 2024, comprised of 29 questions, in multiple-choice question format, aiming to elicit demographic data, diagnostic criteria and treatment modalities of hypotension in preterm infants admitted in Indian NICUs. This was incorporated into an online survey tool (“Google form”- https://www.google.com/forms/about) and tested for content and feasibility by five practising neonatologists. After finalization, the questionnaire was sent to 540 practising neonatal physicians via academic groups on social media platforms. A brief background about preterm hypotension and the need for the survey was provided with the link. Responses in the form of completed questionnaires were considered as consent for participation, and no incentives were offered for participation. All data received was kept confidential. Twice weekly reminders were sent and the survey was closed after two weeks.

### Statistical analysis

Data was collected through Google forms which was then exported into excel sheet. The results of the survey were analysed using descriptive statistics with the SPSS V.25 software. All data points were in qualitative format, which was presented as numbers and percentages. Chi-square test was used to compare the responses of survey participants as per their training status and level of care provided. *P* < 0.05 was considered statistically significant.

## Results

### Demographics of survey participants

A total of 320 neonatal physicians from various neonatal units in India responded to the survey (response rate 59.2%). Of these, 82% (*n* = 263) respondents worked in neonatal units that provided level III care, while the rest (*n* = 57) provided level II or level I care. About 68% of these were formally trained neonatologists, two-thirds of which had more than 5 years’ experience in neonatal care; 10% were paediatricians practising neonatology; while the rest were neonatal trainees. Of the total respondents, 274 physicians specified their hospital affiliation, with a majority (41%) working in private non-teaching hospitals and 32% in private teaching institutions; while 22% worked in public non-teaching hospitals and 5% in public teaching hospitals. The number of preterm admissions per month varied widely between responding physicians. Approximately 61% of physicians reported admitting less than 25 preterm neonates per month in their units, while around 28% admitted between 26 to 50 preterm neonates and 11% admitted more than 50 preterm neonates per month. The distribution of the number of preterm neonates with hypotension and other characteristics of the participating physicians and their neonatal units are depicted in Table 1. Delayed cord clamping (DCC) was practised by 78% of the respondents in their centres.

**Table 1:** Characteristics of survey participants.

Characteristic	Number of respondents (*n* = 320)	Percentage
Level of care provided^a^		
Level I	18	5.6%
Level II	39	12.2%
Level III -A	66	20.6%
Level III -B	155	48.5%
Level III -C	42	13.1%
Role and experience in neonatal care		
Neonatologist >10 years	71	22.2%
Neonatologist 5–10 years	74	23.1%
Neonatologist <5 years	72	22.5%
Pediatrician with special interest in neonatology	32	10%
Trainee	71	22.2%
Bed strength of neonatal unit		
<10	74	23.1%
10–30	182	56.9%
31–50	37	11.6%
>50	27	8.4%
No of preterm admissions per month		
<25	194	60.6%
26–50	90	28.1%
51–100	23	7.2%
>100	13	4.1%
Number of preterm neonates diagnosed with hypotension per month		
0–10	247	77.2%
11–20	44	13.8%
21–30	21	6.5%
>30	08	2.5%

Level I: Basic neonatal care of healthy newborns.

Level II: Specialty care of preterm infants with birth weight <1,500 g.

Level III: Comprehensive neonatal intensive care of ELBW & <28 weeks, with facilities for Ventilator support (III-A), Pediatric surgery (III- B), Cardiac surgery (III -C).

^a^ Levels of care as per accreditation norms of National Neonatology Forum of India (NNF).

### Diagnosis and assessment of hypotension

A little less than half of the participants (44%) reported having a written institutional protocol for the diagnosis of hypotension. The criteria used for diagnosis of hypotension varied and is outlined in Table 2. Around half of the respondents (52%) used “mean BP less than gestational age” as the criteria for diagnosing hypotension in preterm neonates; 38% used various percentile-based BP charts; 9% used an absolute cut-off value of mean BP<30 mmHg; and a small proportion (1.8%) used only indirect clinical criteria such as prolonged capillary refill time, tachycardia etc. About two-thirds of the survey participants (66.9%) used only non-invasive methods to measure BP in preterm neonates with hypotension, while 31% used invasive as well as non-invasive methods. A small proportion (2.2%) used only invasive BP monitoring.

**Table 2:** Blood pressure criteria for defining hypotension in preterm neonates.

Blood pressure criteria	Number of respondents (*n* = 320)	Percentage
Mean BP <GA	165	51.6%
Mean BP <30 mmHg	28	8.8%
Percentile based BP chart (BP <50th centile for GA)	37	11.5%
SBP and DBP <10th centile for GA	49	15.3%
SBP and DBP <3rd centile for GA	28	8.8%
BP <5th centile for GA	04	1.3%
Zubrow's reference curves	03	0.9%
Do not measure BP (use other clinical signs of reduced perfusion such as prolonged CRT, tachycardia, urine output)	06	1.8%

Abbreviations: BP, blood pressure; GA, gestational age; SBP, systolic blood pressure; DBP, diastolic blood pressure; CRT, capillary refill time.

**Figure 1:**
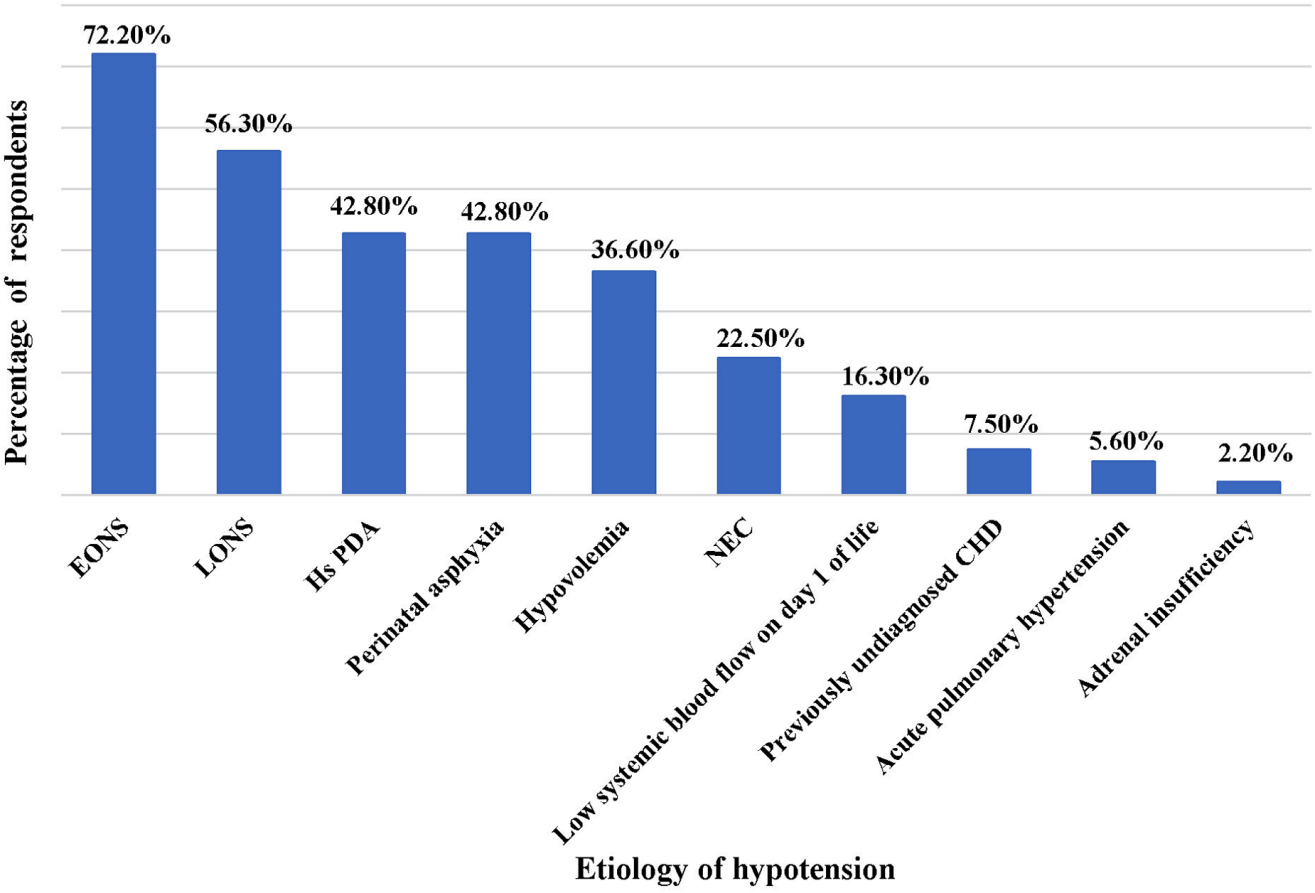
Common causes of hypotension in preterm neonates. EONS, early onset neonatal sepsis; LONS, late onset neonatal sepsis; Hs PDA, hemodynamically significant patent ductus arteriosus; NEC, necrotizing enterocolitis; CHD, congenital heart disease.

The commonest cause of hypotension in preterm neonates observed by the survey participants was early onset neonatal sepsis (EONS), followed by late-onset neonatal sepsis (LONS), hemodynamically significant PDA (Hs PDA) and perinatal asphyxia (Figure 1). The three most important clinical criteria identified by physicians in the evaluation of perfusion were capillary refill time (253 responses), followed by blood pressure (191 responses) and heart rate (178 responses). Of these, capillary refill time (CRT) >3 s was considered by 79% of physicians as one of the more reliable markers of poor tissue perfusion. The common adjunct laboratory criteria utilized for evaluation of hypotension were lactate (253 responses), followed by pH of blood gas (244 responses), and base deficit (229 responses); while markers of myocardial dysfunction such as troponin and brain natriuretic peptide (BNP) were least commonly used.

**Figure 2:**
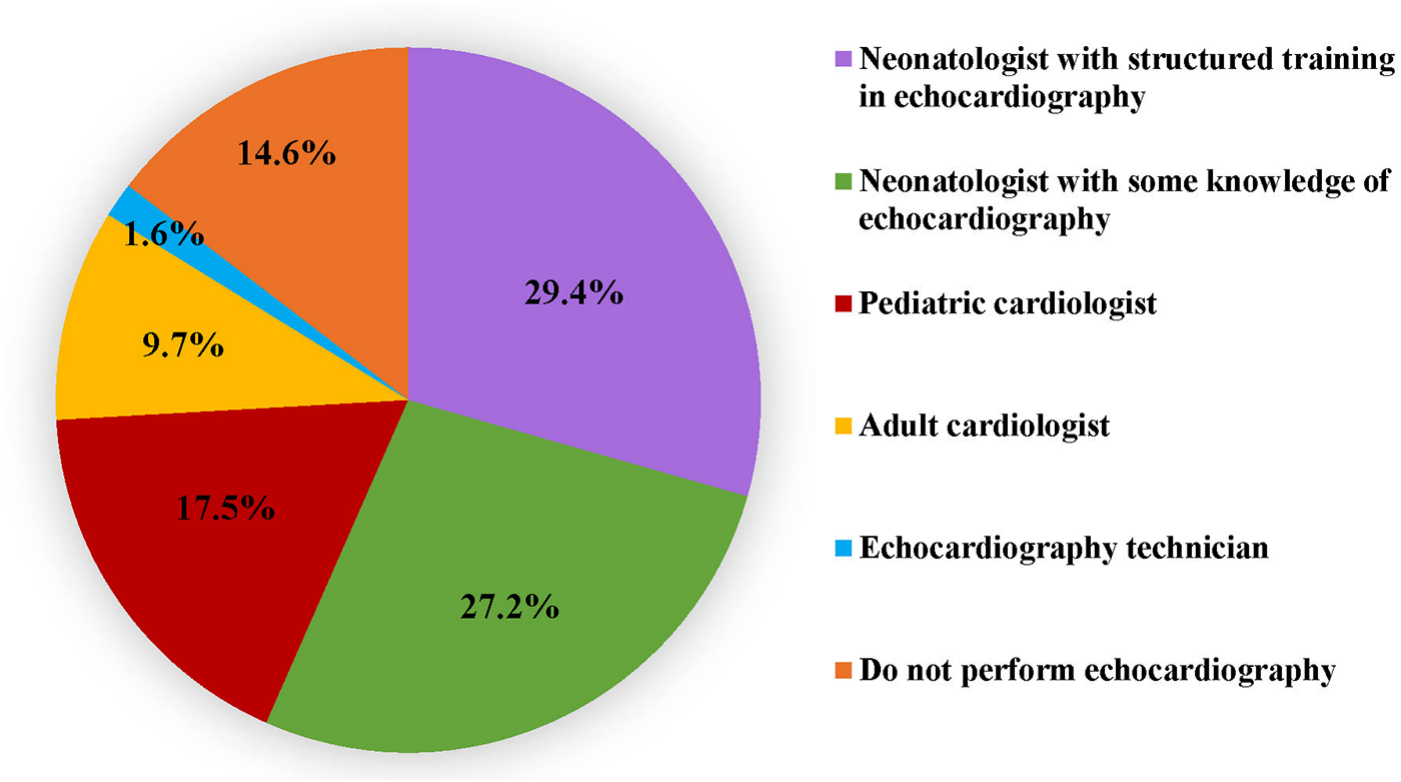
Category of personnel performing echocardiography.

While eighty-five percent respondents utilized echocardiography in the NICU, only 73% utilised it in the assessment of a hypotensive neonate. A little more than half (56.6%) of participants reported that echocardiography was performed in their unit by neonatologists who had undergone a structured training programme (29.4%) or had some knowledge (27.2%) of echocardiography. In the remaining units, echocardiography was performed by paediatric cardiologists, adult cardiologists or echocardiography technicians (Figure 2). Table 3 outlines the various echocardiographic criteria used for evaluation of perfusion in preterm neonates. More than one response was permitted and the commonest criteria used were left ventricular systolic functions (44%) such as fractional shortening (FS), ejection fraction (EF) and left ventricular outputs (LVO); followed by a comprehensive assessment of cardiac functions, filling, cardiac outputs, and pulmonary haemodynamics (39%). Assessment of global cardiac function using myocardial performance index (MPI) was the least used. Only 5% of respondents used advanced technology such as near-infrared spectroscopy (NIRS) to monitor and evaluate cerebral oxygen saturation in hypotension.

**Table 3:** Echocardiography criteria for evaluation of preterm neonate with poor perfusion (multiple responses permitted).

Criteria	Number of respondents (percentage)
Left ventricular systolic functions (such as FS, EF, LVO)	142 (44.4%)
Right ventricular systolic functions (such as TAPSE, FAC, RVO)	72 (22.5%)
Right and Left ventricular diastolic functions (such as E/A ratio)	73 (22.8%)
Global cardiac function (such as MPI)	48 (15%)
Biventricular cardiac outputs	61 (19.1%)
Comprehensive assessment of cardiac function, filling, outputs, assessment of pulmonary hemodynamics	124 (38.7%)
SVC flow	70 (21.9%)

Abbreviations: FS, fractional shortening; EF, ejection fraction; LVO, left ventricular output; TAPSE, tricuspid annular plane systolic excursion; FAC, fractional area change; RVO, right ventricular output; E/A, E-and A- wave velocities; MPI, myocardial performance index; SVC, superior vena cava.

### Treatment of hypotension

The majority of the respondents reported that the treatment initiated as first-line management on detection of hypotension was volume expansion (85%). Around 12% started inotropes as the first line and 3% started vasopressor first.

#### Volume expansion

The initial amount of volume infused by most was 10 ml/kg (90%). Around 7% used 20 ml/kg and a few (3%) used smaller initial volumes of 5 ml/kg. The maximum cumulative volume infused by the majority of respondents was 20 ml/kg (63%), while about 28% gave higher cumulative volumes up to 30 ml/kg and 40 ml/kg. A very small proportion (0.9%) infused more than 40 ml/kg volume. Normal saline was the commonest crystalloid of choice (98%) for volume in preterm neonates with hypotension of non-haemorrhagic aetiology, while 2% used Ringer's lactate solution. None of the respondents reported using colloids such as albumin for non-haemorrhagic hypotension.

#### Inotropes

**Figure 3:**
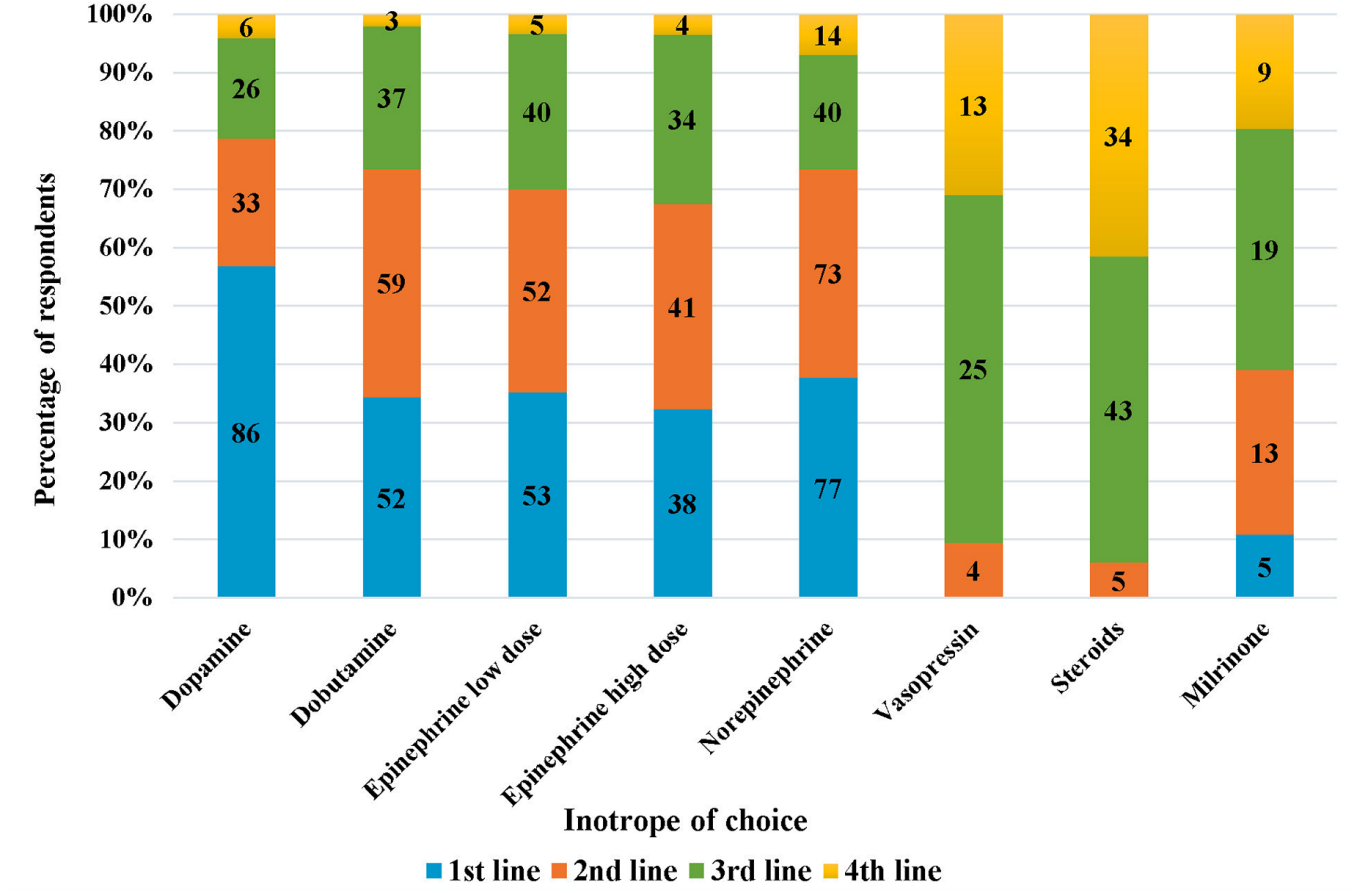
Inotrope of chouce in preterm neonates with hypotension due to sepsis (the numbers inside the bars represent “number of respondents”).

Of those who performed echocardiography for assessment of hypotension, 60% started inotropes first and then performed echocardiography as per convenience, while the rest performed echocardiography before commencement of inotropes**.** On specifically being asked about the inotrope of choice in preterm neonates with hypotension secondary to sepsis**,** dopamine was the most preferred first-line inotrope, followed by norepinephrine and low-dose epinephrine in that order (Figure 3). Norepinephrine, followed by dobutamine was the most preferred second-line agent.

#### Steroids

The commonest steroid used in the treatment of hypotension was hydrocortisone (90%). Around 9% respondents used dexamethasone and a small proportion (1%) used methylprednisolone. The majority (55%) of neonatal physicians in our survey, initiated steroids after they had already started two inotropes (as third-line agent). Around 6% used it as second-line agent (after one inotrope), 26% as the fourth line after three inotropes, and 2.5% as fifth-line agent after multiple inotropes. Only one respondent reported using steroids as a first line agent, and 10% did not use any steroids for treatment of hypotension.

### Variations in management of hypotension between level III and level II + I physicians

**Figure 4:**
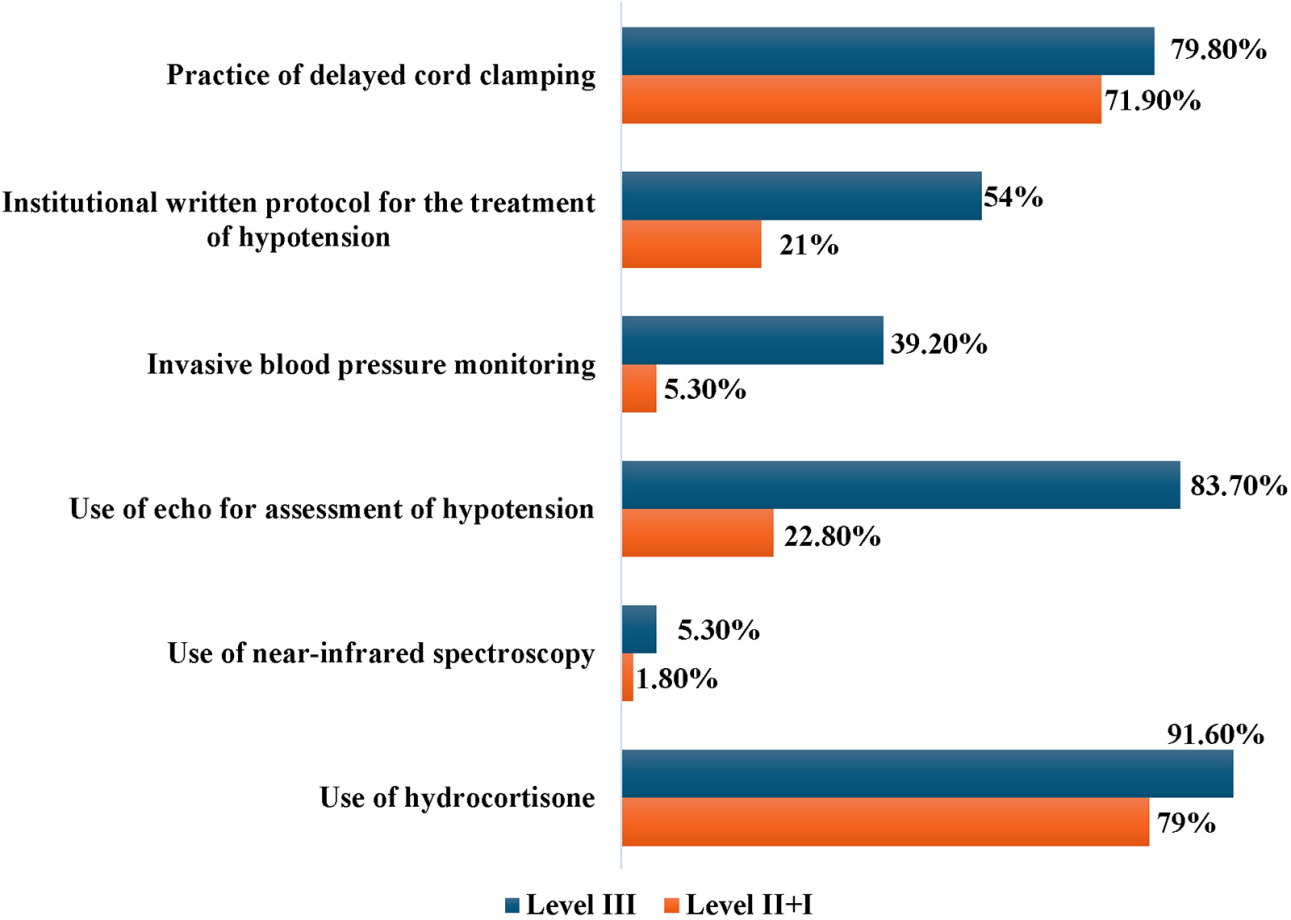
Key variations in the manangement practices between level III vs. level II + I neonatal units.

We compared the responses as per the level of care provided by the survey participants (Figure 4). A description of the levels of NICU care provided in India have been described in Table 1. We found that the majority of trainees (96%) worked in Level III units. The monthly preterm admissions and the number of hypotensive preterm neonates was higher in level III units (*p* < 0.001 and *p* 0.005 respectively). The practice of DCC was uniform irrespective of the level of care provided (level III-79.8%, level II + I-71.9%, *p* 0.188). Level III units were more likely to have an institutional written protocol for treatment of hypotension (*p* < 0.001); however, only 54% level III units had a written protocol. While invasive BP monitoring was predominantly used in level III units, non-invasive BP monitoring was the more commonly used modality in all levels of NICU (Figure 4).

There was no difference in the clinical and laboratory parameters used to evaluate hypotension between the two groups of respondents. A significantly higher number of respondents working in level III units reported use of echocardiography for assessment of a hypotensive neonate compared to level II + I units (83.7% vs. 22.8%, *p* < 0.001). Neonatologist-performed echocardiography was more likely to be performed in level III units, while paediatric cardiologists or adult cardiologists performed echocardiography in lower levels of NICUs (*p* < 0.001). The use of Near Infrared Spectroscopy (NIRS) was very limited, even in Level III NICUs (Figure 4). There was no statistically significant difference in the choice of crystalloid for volume expansion, maximum cumulative volume and the type of fluid given between the groups. The majority of respondents in both groups preferred to use hydrocortisone as steroid of choice for treating hypotension (91.6% and 79% respectively). However, 21% physicians working in level II units reported dexamethasone as the steroidal agent of choice. Respondents working in all levels of NICU reported early onset and late onset sepsis as the commonest causes of hypotension in preterm neonates.

### Variations in hypotension management between respondents depending on training status

We analysed the responses as per training status of the respondents and found a few differences. Of the total respondents in our survey, 77.8% (*n* = 249) were consultants (neonatologists or paediatricians with neonatal interest), while 22.2% (*n* = 71) were trainee physicians working in neonatal units. The majority of trainee respondents were working in Level III units (95.8%) as compared to non-trainees (78.3%) and the differences were statistically significant (*p* 0.0038). In the comparison of the management strategy preferences between the two groups, we found that a higher proportion of trainees (40.8%) preferred to perform an echocardiography before commencement of inotropes as compared to non-trainees (28.5%, *p* 0.0084). There were no statistically significant differences between the two groups in other management strategies.

#### Outcome

The mortality rate due to hypotensive shock in preterm neonates was reported to be <10% by about half of the participants in their respective units. Around 38% reported a rate between 10%–30%, while 9% had a mortality rate of more than 30%; of which 3% had a high mortality rate of >50%. The commonest reported causes of mortality due to hypotension in preterm neonates were late-onset sepsis, followed by early onset sepsis, necrotizing enterocolitis (NEC) and perinatal asphyxia (Table 4).

**Table 4:** Common causes of mortality due to hypotension in preterm neonates.

Cause of mortality	Number (percentage)
LONS	207 (64.7%)
EONS	205 (64.1%)
NEC	140 (43.8%)
Perinatal asphyxia	135 (42.2%)
Previously undiagnosed CHD	67 (20.9%)
Hs PDA	56 (17.5%)
Hypovolemia	39 (12.2%)
Acute pulmonary hypertension	27 (8.4%)
Low systemic blood flow on day 1 of life	26 (8.1%)
Adrenal insufficiency	13 (4.1%)

Abbreviations: LONS, late onset neonatal sepsis; EONS, early onset neonatal sepsis; NEC, necrotizing enterocolitis; CHD, congenital heart disease; Hs PDA, hemodynamically significant patent ductus arteriosus.

## Discussion

In this cross-sectional study, we found wide variation in management practices of hypotension in preterm neonates, among neonatal physicians in Indian NICUs. Previous surveys (5, 6, 12, 13) on hypotension in preterm neonates have addressed populations from high-income nations and there has been no data so far from low-and middle-income countries. We performed a physician-based survey, as opposed to a unit survey, since we wished to ascertain each individual's practice preference.

Our study population largely comprised formally trained neonatologists, or paediatricians with special interest in neonatology, who worked in neonatal units that predominantly provided level III NICU care (average capacity 10–30 beds), and admitting up to 50 preterm neonates per month. However, a few participants (around 11%) belonged to larger units with more than 100 preterm admissions per month. The majority of these units diagnosed up to 20 preterm neonates with hypotension per month, while 10% diagnosed higher numbers. Our data possibly represents the practices of physicians working in units providing an advanced level of care, which could be because the majority of our respondents were formally trained neonatologists, and hypotensive neonates are likely to be referred and managed at higher levels of care. Some previous surveys (6, 12) have assessed the management practices for hypotension during the transitional period (first 24 h of life) in extremely preterm babies using a case-based scenario. In our survey, we adopted a more generic approach, addressing hypotension management in preterm neonates across all gestations.

Delayed cord clamping (DCC) was practised in the units of more than three-fourths (78%) respondents. Several studies have highlighted the potential benefits of placental transfusion in preterm infants, particularly effects on mean BP in the first 24 h; reduced need for inotropes; and effects on cardiac outputs (14–16). DCC is now an essential component of the India Newborn Action Plan (INAP), which is an Indian government initiative to reduce preventable newborn deaths and stillbirths. In a study conducted between 2018 and 2020 (17) in public health facilities in India to explore the practices of DCC, the adoption rate increased to 74% and further to 95% over a span of 2 years (from a baseline of 41%), after capacity building and supported supervision. In our survey, the majority of respondents belonged to private sector NICUs, and the overall rates were comparable to those reported in a recent American survey of obstetricians and neonatal physicians (18) and higher than those reported in surveys of some other countries such as Malaysia (19). It is important to note that while our survey population predominantly consisted of physicians working in advanced levels of NICU care, the practice of DCC was almost uniform irrespective of the level of care provided.

### Clinical criteria

Our survey revealed that most clinical markers including BP were individually deemed unreliable indicators of the cardiovascular status in a neonate. A combination of these markers was considered more reliable, particularly in the critically unwell neonate. A CRT >3 s was individually considered by a majority as one of the more reliable clinical markers of poor tissue perfusion. However, Osborn et al. (20) have reported that CRT and systemic blood flow had a weak association. Studies (5, 20) have also suggest that a combination of clinical parameters and BP data were better indicators of systemic flow, than either alone. In contrast to our study, van Wyk et al. (21) reported that most clinicians (67%) relied on low BP alone, independent of other modalities, for diagnosing neonatal hypotension, but used a combination (BP, clinical, and laboratory parameters) for escalation of hypotension therapy.

More than a third of the physicians in our survey utilised the common adjunct laboratory criteria such as pH, base deficit, and high lactates for the evaluation of hypotension. Miletin et al. suggest that combining criteria like CRT >4 s and serum lactate levels >4 mmol/L had a high specificity (97%) for detecting a low systemic flow state (22). Similarly, other surveys (6, 12) indicate that metabolic acidosis and plasma lactates were considered significant in the evaluation of neonatal condition. In contrast to our study, Stranek et al. (6) reported that 28% respondents considered markers of myocardial function to be significant.

### Blood pressure

One of the primary drivers for the diagnosis of hypotension is BP thresholds, whether measured invasively or non-invasively. An analysis of the surveys conducted in various countries (5, 6, 12, 13, 21) suggests that there are significant variations in the criteria for the diagnosis of hypotension. This is reflected in our survey as well, with approximately half the respondents using the British Association of Perinatal Medicine (BAPM) criteria (7), and the others using varied other thresholds. The recommendations by the Joint Working Group of the BAPM to keep mean BP above the “gestational age in weeks” has minimal evidence to support this guideline, and yet is followed by most physicians. Other surveys (5, 6, 21) variously report that 76%–87% of physicians utilise this criterion. These variations likely stem from several factors, including the challenge of precisely measuring the level at which loss of cerebrovascular auto-regulation occurs; variations in BPs at different gestations and increasing postnatal ages; and differences in invasive vs. non-invasive thresholds. Since these BP values are used as thresholds to commence inotrope/vasopressor therapies, it would be useful to follow standardized criteria laid down by our academic bodies for physicians to minimize variations, particularly since adjunct assessment tools like echocardiography are limited in resource-limited countries. Interestingly, in our survey, 1.8% physicians did not use BP as a criterion to diagnose hypotension at all.

Invasive arterial BP recordings are considered the most accurate method of monitoring BP, particularly in critically sick neonates (23, 24). In the UK survey (13), only 8% used invasive BP monitoring alone, while 92% used both. In our survey, while both invasive and non-invasive BP monitoring was used in level III NICUs, interestingly, a large proportion of level III units used only non-invasive BP recordings. We speculate that this might be due to the cost of equipment for invasive BP monitoring, and also possibly, to avoid complications related to arterial line procedures.

### Echocardiography and NIRS assessment

Nearly 85% of our survey participants reported the general utilization of echocardiography facilities in their units, marking an improvement from the 80% reported in a 2017 survey of point-of-care ultrasound in Indian NICUs (25). However, only 73% of the survey respondents reported that they assessed a neonate with hypotension using echocardiography. This may reflect limited utilization or lack of knowledge of functional echocardiography (FnECHO), despite the availability of an echocardiography facility. Compared to established neonatologists or physicians with neonatal experience, we found that a higher proportion of trainee respondents showed an increased awareness of the role of echocardiography in the assessment of the hypotensive neonate, which in turn, reflects the impact of training in neonatologist-performed echocardiography on young doctors undergoing neonatal training in India. In the international survey of 38 countries (6), the use of echocardiography as an ancillary investigation for evaluation of poor perfusion was about 75%. Similarly in a survey of neonatal units in UK, echocardiography was felt to be a useful tool for the management of hypotension by 75% of participants (13). However, their data reflected practices in 2012 and 2010 respectively, and the utilization of echocardiography has changed considerably in the past decade in these countries. Around 29% of neonatologists in our survey, who performed echocardiography, had undergone structured training in FnECHO, marking a slight improvement from the 25% reported in the earlier survey of POCUS in Indian NICUs (25). Structured training in neonatal FnECHO has progressed in the last few years in India, particularly after the COVID-19 outbreak, with several online academic courses being conducted during the lockdown phase; although later these online courses were combined with hands-on training as well (26). There remains, however, a need for accredited training programs in FnECHO, with a curriculum standardized by national academic bodies such as National Neonatology Forum of India, which also lay out evidence-based clinical practice guidelines for neonatal care at different levels. There is further need to disseminate the utility of echocardiography as an assessment tool for hypotension, to aid the clinician in determining pathophysiology of the disease.

In our survey, only about 5% of respondents utilized advanced technology such as NIRS for evaluation of cerebral or tissue oxygen saturation, and understandably only in level III units. In the international survey of 38 high income countries (6), 15% of respondents used NIRS for evaluation of perfusion, which was much higher than the proportion in our survey, even a decade ago. NIRS is being increasingly utilized in the developed world and has a potential role in monitoring cerebral perfusion in neonates (27). Although we did not explore NIRS further in our questionnaire, we speculate that cost constraints, lack of standards for interpretation, or inadequate expertise could be the reason for non-utilization of this promising technology in Indian NICUs.

### Management strategies

We observed variations amongst different surveys (5, 6, 12, 13) in treatment strategies used for hypotension. Likely reasons include differing perceptions of when therapy is to be initiated, and efficacy of treatment, and possibly the variations in delayed cord clamping practice in preterm vs. term neonates. The overall strategy in management of the hypotension, in our survey, showed that physicians preferred a ‘volume-inotrope-echo-steroid’ sequence, similar to the UK survey (13). Notably, in our survey, only just over half of the level III units reported having a written protocol for the treatment of hypotension in their hospital, indicating a lack of practice uniformity.

A Cochrane review (2001) suggested that the inotrope dopamine was more successful than volume (albumin) in improving hypotension in preterm infants, although neither was better in improving mortality and morbidity in preterm infants. In this review, the trials did not allow any definite conclusions regarding the superiority of volume or dopamine in preterm infants (28). The strategy of ‘permissive hypotension’ pertains to ELBW neonates who exhibit hypotension, but demonstrate evidence of good perfusion, and therefore could remain untreated with inotropes (29). While this strategy is valuable in ELBW neonates to avoid unnecessary treatment, it was not addressed in the questionnaire.

#### Volume administration

Volume expansion is used by a majority of neonatal physicians prior to the commencement of inotropes in the management of hypotension, but its benefits have not been established (30–32). While it is useful in the hypovolemic neonate (with antepartum hemorrhage, vasoactive shock states- sepsis and NEC, and large IVH); it may be detrimental in neonates with PDA as the primary pathophysiology. Besides, cerebral perfusion (assessed by NIRS) has not been seen to improve in poorly perfused preterm infants following volume administration (33). In our study, the majority (85%) used volume as “first line” management, varying from 10 to 40 ml/kg/day before commencing inotropes. Irrespective of aetiology, other surveys have noted variations of volume bolus from 10 ml/kg to 60 ml/kg (5, 6, 21, 34, 35).

#### Inotropes

Several inotropes have been used to treat hypotensive neonates, with no strong evidence to guide the clinician on the choice of drug. A systematic review and meta-analysis comparing drugs (in a pair-wise manner) showed the effectiveness of dopamine in the treatment of neonatal hypotension (36). The UK survey (5) revealed that dopamine was the most commonly used inotrope followed by dobutamine, with epinephrine as the third choice; whereas the Stranak et al. (6) reported that 62% physicians used dopamine as a single drug, and 80% used it in combination with dobutamine. Our study shows that dopamine was the preferred choice, followed by norepinephrine and low-dose epinephrine; which was very similar to the study by van Wyk et al. (21). With the advent and widespread use of echocardiography as an assessment tool, the choice of inotrope could be guided by the underlying pathophysiology (vasodilatory vs. vasoconstrictor physiology) of the disease process, thereby aiding in the choice of inotropes/vasopressors.

#### Steroids

A Cochrane review published in 2011 (37) suggested that steroids were effective in the treatment of refractory hypotension in preterm neonates, with no reported short-term adverse effects. However, long-term safety data was lacking. Steroids used commonly in the NICU include dexamethasone and hydrocortisone, but dexamethasone has long-term neurodevelopmental implications, and hence not recommended (38).

Dramatic variations were noticed in the dosage of steroids varying from the initial (0.1–5 mg/kg) to the maximum dose (0.6–20 mg/kg) (5). Other studies (12, 13) have used steroids as fourth-line agents after volume, and two inotropes. Steroid use was comparatively lower in the South African survey (20%) (21). In our survey, the majority used hydrocortisone as the choice of steroid after inotropes, while a tenth did not use steroids at all.

### Current consensus recommendations in India

The National Neonatology Forum of India (NNF), a professional association of neonatologists, that is actively involved in advocacy, policy-making and capacity-building at different levels of neonatal care, have recently (November 2023) published clinical practice guidelines for the diagnosis and management of shock in neonates (39) Using GRADE methodology, these evidence-based recommendations for management of hypotension and shock have addressed some of the challenges faced by physicians by defining the diagnostic thresholds for treatment of hypotension and shock in preterm and term neonates. However, many of the treatment guidelines related to the choice of inotrope and treatment strategy in specific situations such as cardiogenic shock in perinatal asphyxia and PDA, remain a grey area, with “insufficient evidence to recommend for or against specific inotropic agents or vasopressors as the first-line drug”. Functional echocardiography-based criteria are lacking due to the current gap in training and expertise in the area. However, as indicated by other surveys conducted across Europe, United States, Canada, and other high-income countries, the lack of clinical outcome-based evidence to guide treatment and establishment of a clear correlation between neonatal hypotension and cerebral blood flow/organ perfusion is a worldwide challenge faced by neonatal physicians in the management of hypotension (5, 6, 13). While further research is needed to address these gaps; we expect that the current consensus guidelines of NNF may, to a certain extent, help to standardize some of the variations and dilemmas faced by physicians in Indian NICUs; and guide formulation of unit policies to minimize inter-physician variations in practice.

### Strengths and limitations

To our knowledge, this is the first cross-sectional study that explores physician perceptions, diagnostic criteria and treatment practices of hypotension in preterm neonates among neonatal physicians of India. Responses were received from neonatologists based in 23 different states of India, representing a wide coverage of population geographically. Our questionnaire was fairly comprehensive in covering major aspects of hypotension management practices and the option of free text in many items allowed us to gain useful insights into individual practice preferences of participants. There were several limitations to our study however. Since participation in the survey was voluntary, and given the response rate of just under 60%, the data may not represent the perceptions and practices of all neonatologists in the country. Also, as this was a physician-based survey, the responses reflected personal choices and understanding, and may not reflect unit-based policies. Further, by the very nature of any survey, responses to certain items in the questionnaire, such as estimates of mortality or hypotensive preterm infant volumes may be speculative and subject to recall bias. In order to maintain feasibility, we had to restrict the number of items in the questionnaire, and certain aspects of hypotension-related practices could not be explored, such as management of hypotension in neonates with HsPDA, dosages of inotropes apart from epinephrine, and treatment of hypotension in transitional circulation. Since we did not explore participant responses to volume administration for each etiology individually, it is possible that we might have received different responses for volume expansion as first line treatment. Since this survey addressed hypotension in preterm neonates, the data cannot be extrapolated to term newborns.

## Conclusion

This survey reflects the lack of clinical certainty in the management of preterm hypotension, leading to huge variations in practice in India. The differences in perception of the blood pressure thresholds to be used in a preterm neonate, and the differences in the choice of inotropes are significant. Proactive efforts for raising awareness and dissemination of current national guidelines to facilitate their implementation among practising neonatal clinicians may help to standardize practices. Echocardiography, as an assessment tool, needs to be better utilised to determine pathophysiology and aid in decision-making regarding the choice of inotrope. A formal, structured neonatologist- performed echocardiography training programme which has a competency-based evaluation, accreditation and ongoing assessment has become a necessity. Further research could include outcome-oriented objectives comparing different therapeutic interventions and utilization of technologies such as NIRS, that correlate organ perfusion with hypotension to guide management.

## Data Availability

The original contributions presented in the study are included in the article/Supplementary Material, further inquiries can be directed to the corresponding author.
