# Supplementary material for: A survey on management practices of hypotension in preterm neonates: an Indian perspective
Source: Front Pediatr. 2024 Oct 21;12:1411719. doi: 10.3389/fped.2024.1411719 (PMC11532123; doi:10.3389/fped.2024.1411719)
Supplement: Supplementary file 1 [file Datasheet1.pdf]

PROFORMA

“A survey on management practices of hypotension in preterm neonates: An Indian perspective”

Background:

The purpose of this study is to evaluate the current perceptions and practices among physicians in the diagnosis and management of hypotension in preterm neonates, admitted in Indian NICUs. We are grateful that you have participated in the survey. The individual’s identity will remain confidential through the data collection and analysis.

Background and Unit Demographics

1. Name/ Position of the person filling out this survey
2. Name of your Institution/City/State
3. What level of care does your NICU provide (as per NNF accreditation norms):
    - Level 1: Near-term and term neonates, Newborn Nursery
    - Level 2: Level 1+Neonates <1500g, Special Care nursery
    - Level 3A Comprehensive care of neonates <28 weeks and ELBWs, provision of ventilator support (Level 3A)
    - Level 3B (Level 3A + pediatric surgery)
    - Level 3C (Level 3A + Level 3B + cardiac surgery).
4. What is your role in the NICU:
    - Consultant neonatologist with >10 years’ experience in neonatal care
    - Neonatologist with >5 and <10 years’ experience in neonatal care
    - Neonatologist with <5 years’ experience in neonatal care
    - Fellow in Neonatology (NNF, IAP, DM, or DNBE Neonatology)
    - Others (please specify):
5. What is the bed strength of your NICU?
    - < 10 beds
    - 10-30 beds
    - 31-50 beds
    - >50 beds

6. What is the total number of preterm neonates admitted per month to your NICU?
    - < 25/month
    - 26-50/month
    - 51-100/month
    - >100/month
7. What is the total number of preterm neonates diagnosed with hypotension in your NICU per month?
    - 0-10/month
    - 11-20/month
    - 21-30/month
    - >30/month
8. Is delayed cord clamping routinely practiced in the delivery room of your hospital?
    - Yes
    - No

Patient Identification, diagnosis, and Screening

9. Does your NICU have an institutional written protocol for the diagnosis of hypotension in the neonate?
    - Yes
    - No
10. Which blood pressure criteria do you use to define hypotension in a neonate?
    - Mean BP less than the gestational age (GA) (as defined by the British Association of Perinatal Medicine)
    - Mean BP < 30 mmHg
    - Using a percentile-based BP chart, <50th centile for the GA
    - If systolic (SBP) and diastolic BP (DBP) is < 10th centile for the GA
    - If the SBP and DBP is < 3rd centile for the GA of the neonate
    - Others(please specify):
11. How do you measure BP in a preterm neonate with hypotension?
    - Invasive monitoring only (arterial lines only)
    - Non-invasive monitoring (BP cuff)
    - Both invasive and non-invasive monitoring
12. What are the 3 **most common causes** of hypotension in preterm neonates in your NICU?
    - Early onset neonatal sepsis (EONS)

- Late onset neonatal sepsis (LONS)
- Previously undiagnosed congenital heart disease
- Hypovolemia
- Necrotizing enterocolitis (NEC)
- Acute Pulmonary hypertension
- Patent ductus arteriosus (Hs PDA)
- Perinatal asphyxia
- Low systemic blood flow on Day1 of life
- Adrenal insufficiency

13. Amongst the enumerated clinical parameters used to evaluate perfusion, please indicate 3 in order of importance which you think are important in the diagnosis of hypotension in preterms:
    - Skin color
    - Capillary Refill Time>3 secs
    - Heart rate
    - Pulse volume
    - Urine output
    - Activity
    - Blood pressure
14. Which of the following lab parameters do you use in your NICU for evaluation of hypotension in a preterm neonate? (please select in order of importance)
    - pH of blood gas
    - Base deficit
    - Lactates
    - Markers of myocardial dysfunction (Troponin, BNP)
15. Does your unit assess a hypotensive preterm neonate with an ‘Echocardiography”?
    - Yes
    - No
16. If an echocardiography is undertaken, who performs the echo?
    - Adult cardiologist
    - Pediatric cardiologist
    - Echocardiography technician
    - Neonatologist with some knowledge of functional echocardiography
    - Neonatologist who has completed structure training in echocardiography

- We do not perform echocardiography

17. What are the echocardiographic criteria you use to evaluate a preterm neonate with poor perfusion? (More than one option can be selected)
    - SVC flow
    - Right ventricular systolic functions like TAPSE, FAC, RVO
    - Left ventricular systolic functions like FS, EF, LVO
    - Right and left ventricular diastolic functions like E/A ratio
    - Global cardiac function using the Myocardial Performance Index
    - Biventricular cardiac outputs
    - Comprehensive assessment of cardiac function, filling, outputs and assessment for pulmonary hypertension
    - We do not use echocardiography in our unit
18. Do you use NIRS as a modality to monitor and evaluate cerebral oxygen saturation in your NICU?
    - Yes
    - No

Management principles

19. Does your NICU have an institutional written protocol for the ***treatment*** of hypotension in preterm neonates?
    - Yes
    - No
20. What treatment do you initiate as first-line management, if hypotension is detected?
    - Volume expansion
    - Inotropic support
    - Vasopressor support
    - Steroids
21. If you use volume, how much volume do you infuse initially?
    - 10 ml/kg
    - 20 ml/kg
    - Others, please specify
22. What is the maximum cumulative volume that you would infuse?
    - 10 ml/kg
    - 20ml/kg
    - 30ml/kg

- 40ml/kg
- >40 ml/kg

23. Which is your crystalloid/colloid of choice for volume in preterm neonates with hypotension due to non-hemorrhagic etiology?
    - Normal Saline
    - Albumin
    - Ringer lactate
    - Others (please specify)
24. Do you commence inotropes before echocardiography in preterm neonates with hypotension?
    - We do not have facility for functional echocardiography in our unit
    - We first perform echocardiography and then commence inotropes
    - We start with inotropes and then perform echocardiography as per convenience
25. In preterm neonates who have hypotension due to sepsis, which is your preferred inotropic agent of choice for circulatory support (in order of preference)?
    - Dopamine
    - Dobutamine
    - Epinephrine low dose (0.01-0.1 mcg/kg/min)
    - Epinephrine high dose (0.1-1.0 mcg/kg/min)
    - Norepinephrine
    - Vasopressin
    - Steroids
    - Milrinone
26. If you use steroids to correct hypotension, which agent do you use?
    - Dexamethasone
    - Hydrocortisone
    - Methylprednisolone
27. When do you use steroids for hypotension in preterm neonates?
    - Steroid is used as first agent of choice
    - One inotrope followed by steroid
    - Two inotropes followed by steroid
    - Three inotropes followed by steroid
    - Four inotropes followed by steroid

- We do not use steroid in our unit

28. What is the mortality rate among preterm neonates diagnosed with hypotensive shock in your unit?
    - < 10%
    - 10%-30%
    - 31%-50%
    - >50%

29. What are the 3 commonest causes of mortality due to hypotension in preterm neonates in your unit?
    - Early onset neonatal sepsis (EONS)
    - Late onset neonatal sepsis (LONS)
    - Previously undiagnosed congenital heart disease
    - Hypovolemia
    - Necrotizing enterocolitis (NEC)
    - Acute Pulmonary hypertension
    - Patent ductus arteriosus (HsPDA)
    - Perinatal asphyxia
    - Low systemic blood flow on Day1 of life
    - Adrenal insufficiency
